# Imaging Findings of an Unusual Malignant Renal Tumor in an Infant

**DOI:** 10.7759/cureus.74519

**Published:** 2024-11-26

**Authors:** Shadi Daghighi, Amr Wardeh, Hatem Al Kashroom, Amy Brady, Daniel Zaccarini, Ravikumar Hanumaiah, Anand Majmudar

**Affiliations:** 1 Radiology, State University of New York Upstate Medical University, Syracuse, USA; 2 Pathology, State University of New York Upstate Medical University, Syracuse, USA

**Keywords:** abdominal distension, aggressive neoplasm, epithelial, malignant rhabdoid tumor, palpable abdominal mass

## Abstract

Malignant rhabdoid tumor is a rare highly aggressive neoplasm that affects young children. It is composed of stromal and epithelial components and commonly arises from the kidney. The clinical presentation is usually nonspecific, and the common signs are palpable abdominal mass, hematuria, fever, anemia, and hypercalcemia. Here we present a case of malignant rhabdoid tumor presenting with anorexia and abdominal distention.

## Introduction

In the intricate landscape of pediatric oncology, rhabdoid tumors emerge as a particularly aggressive and rare entity, arising from primitive cells within the renal medulla [1,2]. Despite their rarity, these tumors wield a substantial impact, accounting for less than 2% of all childhood renal tumors [1,2]. With a distinct predilection for the early stages of life, rhabdoid tumors often make their presence known at an average age of 11 months, frequently affecting male infants [3,4]. The clinical presentation of these tumors is marked by a constellation of symptoms, including abdominal distention, palpable abdominal mass, gross hematuria, and fever [3-5]. A notable aspect of rhabdoid tumors is their propensity to predominantly target a single kidney, albeit their manifestation can extend beyond the renal confines [3]. It's notable that, at the point of diagnosis, a significant portion of afflicted patients already face advanced disease, with more than two-thirds grappling with metastases, most frequently spreading to the lungs and brain [3,4].

## Case presentation

**Figure 1:**
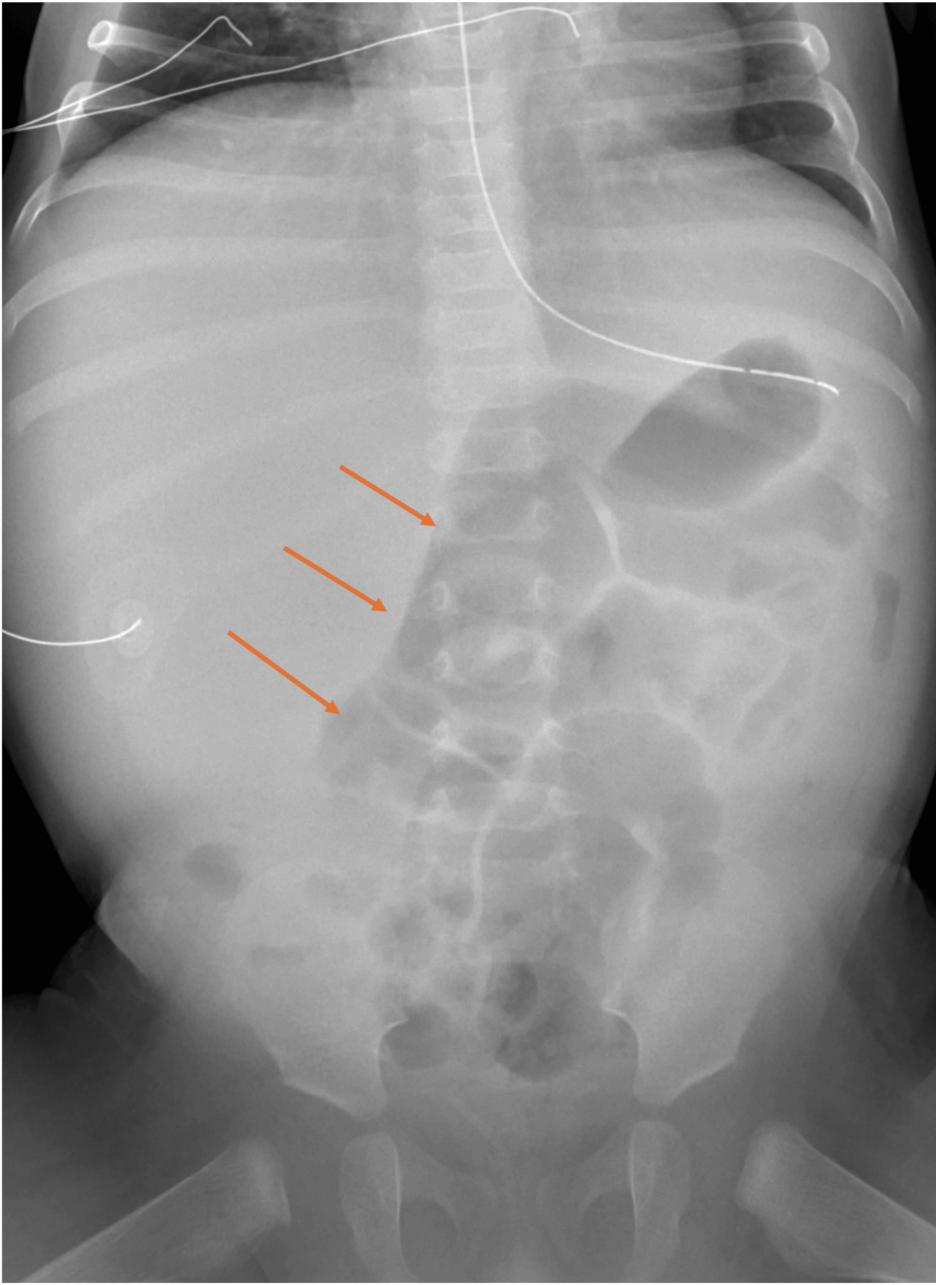
Supine Abdominal radiograph demonstrates displacement of the bowel loops to the center and left abdomen (orange arrows)

A seven-month-old male born following an uncomplicated pregnancy, suddenly developed constipation, fever, abdominal distention and anorexia. The patient had been intermittently febrile with no associated rashes, conjunctivitis, cough, or rhinorrhea. Due to persistent fevers and decreased oral intake, constipation, emesis with brownish material and distended firm abdomen, he was brought to the hospital. His initial workup demonstrated leukocytosis with neutrophilia, lymphocytosis, monocytosis, bands and immature granulocytes, microcytic anemia, significant metabolic acidosis, hyponatremia, acute kidney injury (AKI), elevated transaminitis (aspartate aminotransferase (AST) > alanine aminotransferase (ALT)), hypoalbuminemia, hyperuricemia, elevated lactate dehydrogenase (LDH) >2500; elevated creatine kinase (CK) 5000, free T4 (FT4), C-reactive protein (CRP) and procalcitonin. He also had proteinuria, hematuria, and pyuria, elevated human chorionic gonadotropin (hCG), AFP (alpha-fetoprotein) 667, international normalized ratio (INR) 2.2 and partial thromboplastin time (PTT). The initial abdominal radiograph demonstrated displacement of the bowel loops to the center and left abdomen (Figure 1).

**Figure 2:**
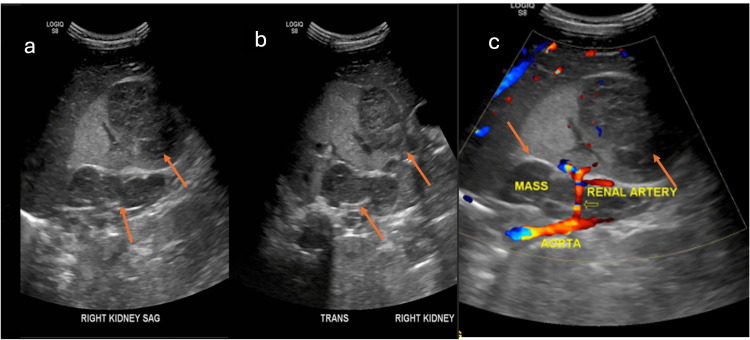
Sagittal and transverse (a,b) of the right kidney shows a well-circumscribed heterogeneous predominantly hypoechoic lesion in the lower pole of the right kidney with another hypoechogenic lesion projecting in the retroperitoneal region along the medial aspect of the right kidney. (c) Doppler image shows no definite vascularity within the lesion (orange arrows).

Initial abdominal ultrasound demonstrated a well-circumscribed heterogeneous predominantly hypoechoic lesion in the lower pole of the right kidney with another hypoechoic lesion projecting in the retroperitoneal region along the medial aspect of the right kidney, and an irregular hypoechoic lesion adjacent to the left kidney. These lesions did not demonstrate significant vascularity (Figure 2).

**Figure 3:**
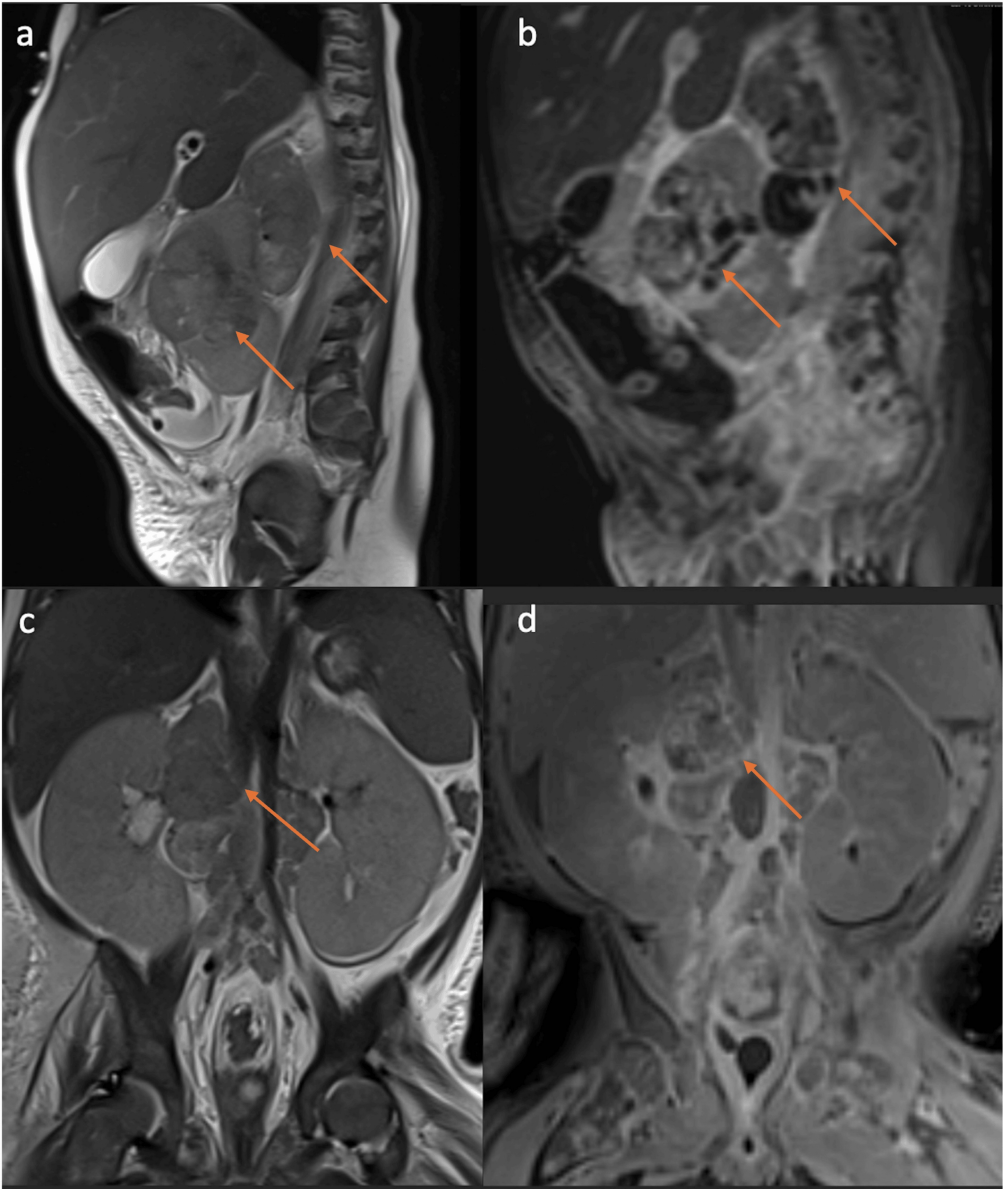
(a) Sagittal T2 image shows heterogenous, predominately T2 hypointense right renal mass (orange arrows). (b) Post-contrast sagittal T1 shows heterogeneous enhancement of the renal right renal mass (orange arrows). (c) Coronal T2 shows extensive bulky retroperitoneal adenopathy (orange arrow) and enlarged bilateral kidneys. (d) Post-contrast coronal T1 shows subtle enhancement within the retroperitoneal lymph nodes (orange arrow).

MRI of the abdomen and pelvis demonstrated large heterogenous, predominately T2 hypointense right renal mass with additional extensive bulky retroperitoneal adenopathy and enlarged bilateral kidneys (Figures 3A, 3C). On postcontrast images, there was subtle enhancement within the retroperitoneal lymph nodes (Figures 3B, 3D). 

**Figure 4:**
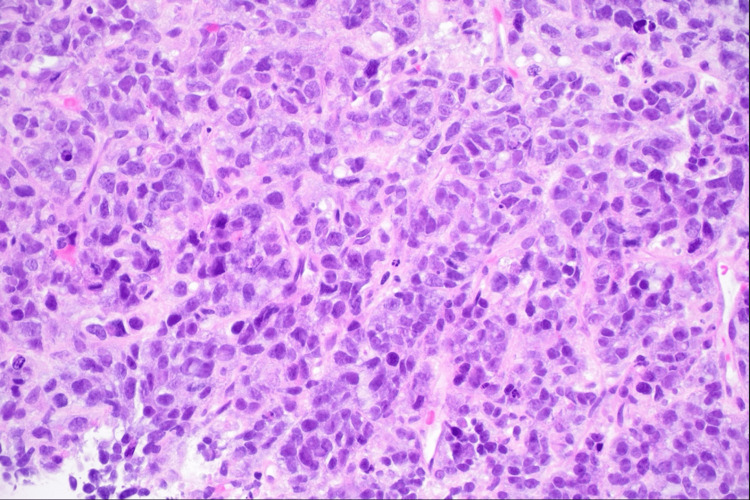
Photomicrograph shows the core biopsy specimen on H&E at 400x magnification. There are sheets of tumor cells with large, high-grade nuclei and eosinophilic cytoplasm within a network of fibrovascular septa. The intracytoplasmic, eosinophilic, hyaline inclusions typically seen in this tumor type are not readily visible

Subsequently, an interventional radiology (IR)-guided right renal mass core biopsy was performed. The biopsy result showed an undifferentiated malignant round cell neoplasm with areas of necrosis, favoring malignant rhabdoid tumor (Figure 4).

**Figure 5:**
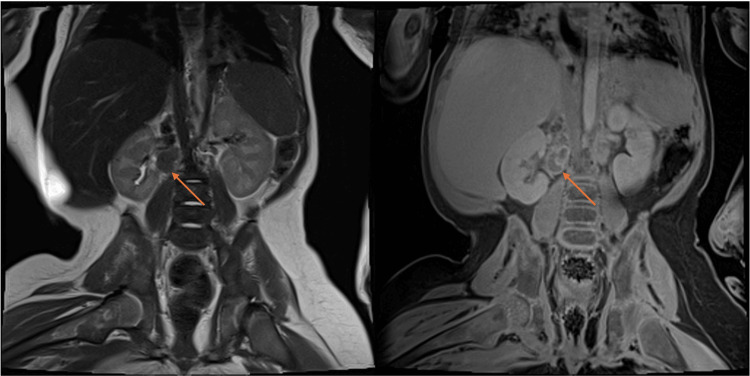
Post-treatment MRI: coronal T2 (left) and coronal T1 post-contrast on the right demonstrate positive treatment response and decreased size of the mass (orange arrows).

**Figure 6:**
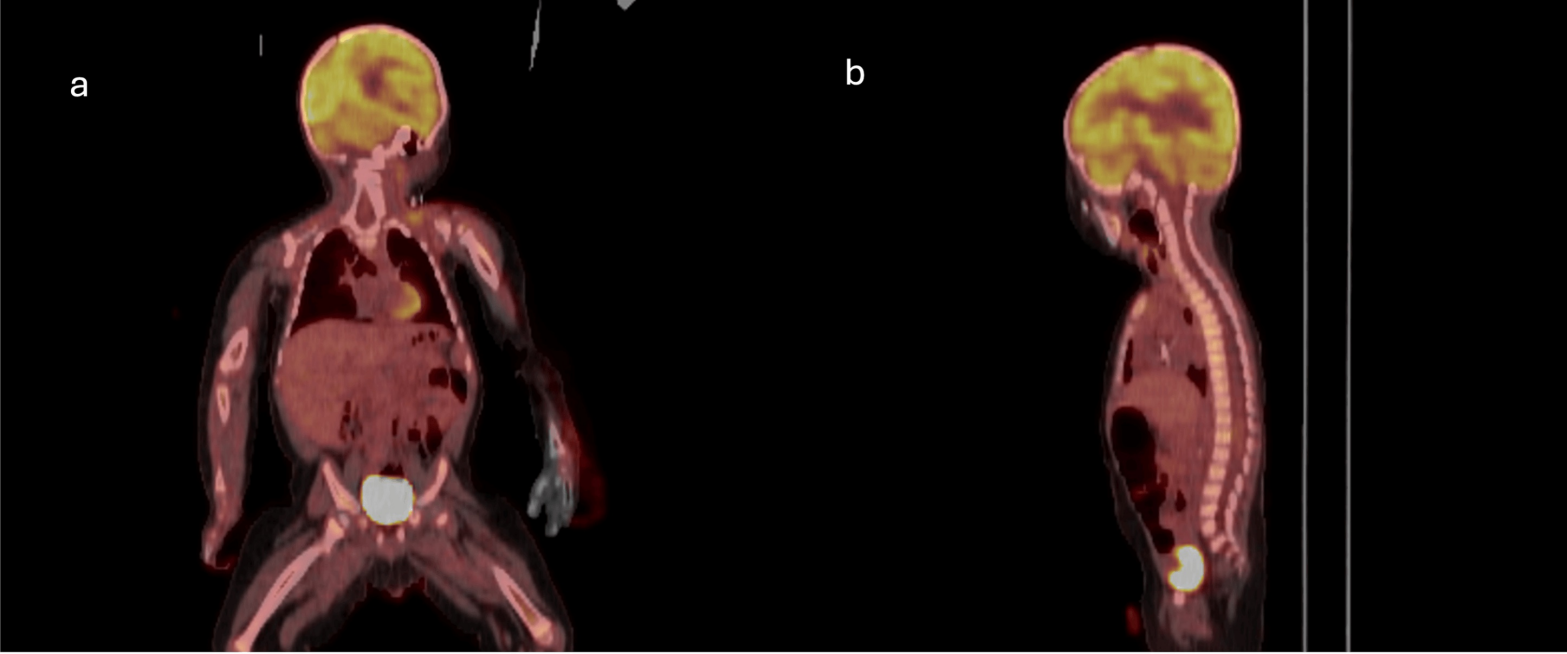
Coronal (a) and Sagittal (b) PET/CT demonstrates no distant metastasis.

The differential diagnosis included Wilms tumor, clear cell sarcoma of the kidney and, less likely, an Ewing sarcoma family of tumors. Immunohistochemistry (IHC) revealed tumor cells negative for AE1/AE3, PAX8, OCT4, CD34, SALL4, chromogranin, synaptophysin, NGFR CD20, WT1, cyclin D1, and SATB2, with lost expression of BRM (SMARCA2) and retained expression of INI1 (SMARCB1) and BRG (SMARCA4). Pan-NTRK showed patchy staining. Fluorescence in situ hybridization (FISH) was performed on the specimen and it was negative for EWSR1 gene break-apart rearrangement. Archer FushionPlex (Integrated DNA Technologies, Inc. Coralville, IA, USA) showed no evidence of gene fusions. There was no mRNA upregulation of BCAR and ETV1/4/5 by manual inspection. The WT1 immunostain was negative, making Wilms tumor less likely, and FISH for EWSR1 gene break-apart rearrangement was negative, making Ewing sarcoma family of tumors less likely. Cyclin-D1 and SATB2 show diffuse positivity in clear cell sarcoma of the kidney, but they were negative in this case. A complete loss of BRM (SMARCA2) expression and a retention of INI1 (SMARCB1) in the tumor cells was suggestive of unusual malignant rhabdoid tumor. After the initial diagnosis, treatment was immediately started with vincristine, cyclophosphamide, and doxorubicin for a five-day course. Vincristine was also given on days 8 and 15 of treatment. Follow-up MRI performed one month later demonstrated significant interval decrease in the size of right renal mass and interval decrease in size of the kidneys bilaterally (Figure 5). The mesenteric lymphadenopathy completely resolved. Retroperitoneal and renal hilum lymphadenopathy significantly improved. PET imaging was obtained and demonstrated no distant metastasis (Figure 6). Due to the patient’s favorable response to therapy and significant decrease in tumor size, a second cycle of vincristine, cyclophosphamide and doxorubicin was initiated. Afterwards, a right nephrectomy and lymphadenectomy were performed approximately two months after the initial presentation. The patient tolerated the procedure well and had no postoperative complications.

## Discussion

The rhabdoid tumors are rare and aggressive renal neoplasm arising from primitive cells within the renal medulla, rhabdoid tumors constitute a mere fraction, less than 2%, of childhood renal tumors, yet their impact is profound [1,2]. The relatively tender age at which these tumors manifest, with a mean age of 11 months and a notable male predominance, underscores the urgency of understanding their clinical behavior and presentation [3,4].

The presenting symptoms of rhabdoid tumors are abdominal distention, palpable abdominal masses, gross hematuria, and fever which requires the need for early detection and intervention [3,5]. 

An intriguing aspect of rhabdoid tumors is their predilection for CNS and kidneys, with most cases affecting a single kidney [3]. This propensity for unifocal presentation sets them apart from other renal tumors, further influencing their clinical course and management considerations. Although there was no distant metastasis in our case, however, the prognosis is often clouded by the stark reality that, upon presentation, more than two-thirds of patients face advanced disease, characterized by the ominous spread of metastases to the lungs and brain [3,4]. This daunting scenario emphasizes the aggressive nature of these tumors and highlights the challenges associated with their treatment.

Diagnostic imaging has an important role in the understanding and management of rhabdoid tumors. Computed tomography usually demonstrates a heterogeneous mass, often accompanied by areas of necrosis, often revealing large, poorly defined masses with possible calcifications and hemorrhage, which are common imaging characteristics. On MRI, rhabdoid tumors are known for their aggressive nature, with features like high signal intensity on T2-weighted images and low to intermediate signal on T1-weighted images. Diffusion-weighted imaging (DWI) often shows restricted diffusion due to high cellularity. Additionally, these tumors can display contrast enhancement, especially in areas of necrosis or hemorrhage [6].

In renal rhabdoid tumors, common findings include unencapsulated, heterogeneous masses with frequent calcifications and subcapsular hemorrhage, however these were not seen in our case, which makes the case an unusual presentation. The tumors often invade adjacent structures, such as the renal hilum, making them appear invasive on imaging [7]. The presence of renal capsular thickening, peripheral subcapsular fluid collection, and calcifications within the tumor adds additional dimensions to their radiological portrait. Particularly noteworthy is the consistent finding of a peripheral collection, observed in a substantial majority of cases, thus serving as an important finding helping with the diagnosis of rhabdoid tumors [1,2]. These imaging features are critical for differentiating rhabdoid tumors from other pediatric malignancies, although histopathological confirmation is necessary for a definitive diagnosis.

## Conclusions

In conclusion, the discussion on rhabdoid tumors unveils their aggressive behavior. Their non-specific clinical presentation and their radiological features can aid in their identification. As a rare yet impactful entity in pediatric oncology, rhabdoid tumors emphasize the importance of early diagnosis, multidisciplinary collaboration, and innovative therapeutic strategies to counteract their aggressive nature and improve patient outcomes.
